# Spatial accessibility of emergency medical services in Chongqing, Southwest China

**DOI:** 10.3389/fpubh.2022.959314

**Published:** 2023-01-06

**Authors:** Yang Zou, Ling Jia, Saijuan Chen, Xinyi Deng, Zhiyi Chen, Ying He, Qiuting Wang, Dianguo Xing, Yan Zhang

**Affiliations:** ^1^School of Public Health, Research Center for Medicine and Social Development, Innovation Center for Social Risk Governance in Health, Research Center for Public Health Security, Chongqing Medical University, Chongqing, China; ^2^Office of Health Emergency, Chongqing Municipal Health Commission, Chongqing, China

**Keywords:** emergency medical services, nearest neighbor method, spatial accessibility, spatial pattern, Chongqing

## Abstract

**Background:**

Timely access to emergency medical services (EMS) can significantly reduce mortality. In China, the evidence of the accessibility of complete EMS which considers two related trips and involves large rural areas is insufficient. This study aimed to explore the accessibility of ambulance services and complete EMS in Chongqing and its regional differences, and to provide a reference for improving spatial accessibility of EMS in Chongqing and optimizing allocation of EMS resources.

**Methods:**

The nearest neighbor method was used to measure spatial accessibility of ambulance services and complete EMS. Spatial aggregation patterns and influencing factors of spatial accessibility of complete EMS were analyzed using Moran's I index, Pearson correlation and multiple linear regression.

**Results:**

The medians of shortest travel time for ambulance, monitoring ambulance, primary EMS and advanced EMS in Chongqing were 7.0, 18.6, 36.2, and 47.8 min. The shortest travel time for complete EMS showed significant spatial aggregation characteristics. The Low-Low types that referred to cluster of short EMS travel time mainly distributed in city proper. The High-High types that referred to cluster of long EMS travel time mainly distributed in northeast and southeast of Chongqing. Urbanization rate was a negative influencing factor on shortest travel time for primary EMS, while average elevation and the number of settlements were positive influencing factors. GDP per capita and urbanization rate were negative influencing factors on shortest travel time for advanced EMS, while the number of settlements was a positive influencing factor.

**Conclusion:**

This study evaluated the accessibility of EMS which considers two related trips in Chongqing. Although the accessibility of ambulances in Chongqing was relatively high, the accessibility of monitoring ambulance was relatively low. Regional and urban-rural differences in the accessibility of complete EMS integrating two related trips were obvious. It was recommended to increase financial investment in economic backward areas, increase high-quality EMS resources, enhance EMS capacity of central township health centers, strengthen road construction in mountainous areas, and provide reasonable planning of rural settlements for improving the spatial accessibility of EMS, narrowing the urban-rural gap and improving equity in getting EMS for all the people.

## Introduction

Emergency medical services (EMS), as an important part of the healthcare system, provides pre-hospital emergency care and medical transport for patients in the case of medical emergent events (1). EMS plays a critical role in saving patients' lives and improving health outcomes. Studies have shown that timely and effective EMS could reduce unintentional injury deaths by 80% in adults and 60% in children (2, 3) and could reduce mortality by 45% in low- and middle-income countries (4). However, the scarcity and uneven distribution of EMS resources remains a critical health problem, especially in low- and middle-income countries and economic backward regions (5, 6). Evidence pointed out that rural residents often faced delays in accessing pre-hospital care and being transported to hospitals compared with urban residents (7). To meet the global demand for EMS and to improve equity in access to EMS, the World Health Assembly (WHA) adopted Resolution 60.22 in 2007, proposing “*to assess comprehensively the prehospital and emergency-care context including, where necessary, identifying unmet needs”* (8). Another 2015 proposal suggested that by 2030, at least 80% of the population in any country should have access to emergency surgery in order to achieve the international goal of access to essential health services and to increase the accessibility of EMS (9).

Essentially, accessibility is a method of how easy it is for users to access public services (10). From a utilization perspective, accessibility can be divided into two categories: revealed accessibility and potential accessibility (11), with the former focusing on actual use of EMS and the latter emphasizing the provision of EMS. From a spatial perspective, spatial accessibility usually refers to the travel impedance (e.g., travel distance or travel time) between an emergency facility and a patient (11); the non-spatial perspective focuses on social, demographic, economic, and health policy factors that may influence the difficulty degree of accessibility of EMS (12). This study was interested in the potential spatial accessibility of EMS.

Over the past decades, many indicators have been developed to measure potential spatial accessibility, which can be classified as provider-to-population ratios, travel impedance-based measures, gravity model and so on (13, 14). Since “time is life,” the time required to arrive at the accident site and transfer the injured to the hospital is considered as the key performance indicator of EMS (15, 16), and travel time is often used to estimate accessibility, especially in larger study regions (6, 17, 18). Furthermore, in remote rural areas where residents have poor health service selectivity, travel time to the nearest medical institution is considered as a good measure of accessibility in rural areas (13, 19), which can capture the proximity between residents and the location of medical service (20) and directly reflect the difficulty of seeking medical care for rural residents (21). Therefore, time to the nearest provider is widely used to measure the spatial accessibility of EMS.

Studies on the spatial accessibility of EMS have been conducted in several countries and cities, but there are still some limitations: First, existing studies usually considered only one-way trip time interval (6, 17, 18, 22–24). But a complete EMS process usually involves two related trips of ambulance. The first time is that the EMS station sends an ambulance to the accident site, and the second time is that the ambulance transfers patient from the accident site to the rescue institution. Depending on patient's condition, the patient may be transported to different facility, so the time interval between the two trips may vary greatly. Therefore, integrating the two-trips time intervals can more accurately reflect spatial accessibility of EMS. Second, most of study areas were relatively narrow and limited to one city usually, including urban central areas and suburbs (24–29). Studies in China, in particular, focused on economically developed cities such as Beijing (30), Shanghai (31), and Wuhan (32). China's New Medical Reform in 2009 incorporated EMS into the public health service system (33). For the recent more than a decade, China government has promoted the construction of a rural EMS system (34) and established the goal of building a government-led EMS system covering both urban and rural areas which adapts with the social economy development by 2025 (35). What exactly is the status of the construction? There have been few studies on the EMS accessibility in rural areas and comparisons of the urban-rural disparity. Only one study from Sichuan province involved a large rural area, but it calculated the time that residents getting to the nearest emergency facility on their own and did not consider the availability of ambulance services in EMS (18). Third, the configuration of ambulances varies greatly between regions, especially in resource-limited areas. For example, in China, ambulances in rural areas are mostly transit-type ambulances equipped with only simple equipment such as stretchers and oxygen bags, while ambulances in urban areas are mostly monitoring-type ambulances equipped with equipment of emergency care such as ventilators, cardiac monitors and defibrillators. Different types of ambulances resulted in different emergency care outcomes (36). But current accessibility studies did not consider ambulance type and class (32). Finally, most studies have focused only on descriptive analysis of the spatial distribution of EMS accessibility (6, 29, 32, 37) and lacked a more comprehensive factor analysis of the socio-economic, geographic and population factors affecting EMS accessibility.

Chinese EMS system began in the 1950's and has developed rapidly since the 1980's, with various EMS modes established in various regions gradually. The “independent” EMS mode was gradually formed in economically developed large cities such as Beijing and Shanghai, where the EMS network consisted of emergency centers and EMS stations (38). According to EMS demand, emergency centers were planned in a unified manner and were mainly responsible for pre-hospital emergency services, and patients were transported to medical institutions with treatment capability for in-hospital emergency services after pre-hospital resuscitation processing (38). The less economically developed central and western regions of China, especially the vast rural areas, mostly adopted the “dependent” EMS mode. In this mode, emergency centers and EMS stations were established by relying on hospitals at all levels and capable primary care institutions, which had both pre-hospital and in-hospital EMS functions (39).

Chongqing, located in southwestern China, is a typical mountainous city with a dual economic structure of large city and large rural area. Chongqing is a typical representative city of the “dependent” EMS mode in China (40). Chongqing has established a unified 120 dispatching and command system covering the whole city, including one municipal dispatching center and 31 district/county dispatching sub-centers, dispatching the whole city's emergency medical resources. Due to the economic and resource constraints, especially the limited health manpower in the large rural areas, EMS stations in Chongqing were established by relying on all levels of hospitals and primary medical institutions with emergency capabilities to provide EMS and obeyed the unified dispatching of the dispatching center. Primary care institutions (including community health service centers and township health centers) and level I hospitals mainly provide pre-hospital emergency services for the surrounding residents and in-hospital emergency services for patients with mild symptoms. Patients with severe conditions are often sent to secondary and above hospitals. The secondary and tertiary hospitals have specialized emergency departments, intensive care units, etc., which can provide in-hospital treatment for patients with acute and critical illnesses, in addition to pre-hospital emergency services for the surrounding residents. Tertiary hospitals have more comprehensive performance and can provide the multiple-discipline comprehensive treatment for patients with more complex condition. At present, there is still a lack of analysis of spatial accessibility of EMS in the less economically developed central and western regions of China, especially the vast rural areas, where the “dependent” EMS mode is adopted.

In this study, we intended to analyze the spatial accessibility of EMS in Chongqing and its urban-rural and inter-regional disparities based on the shortest travel time. We also attempted to explore the factors that influenced the spatial accessibility of EMS from socio-economic, medical resources, geography and population aspects, with a view to providing a basis for improving the spatial accessibility of EMS in Chongqing, optimizing the allocation of EMS resources, and providing reference for other similar regions.

## Materials and methods

### Study area

**Figure 1:**
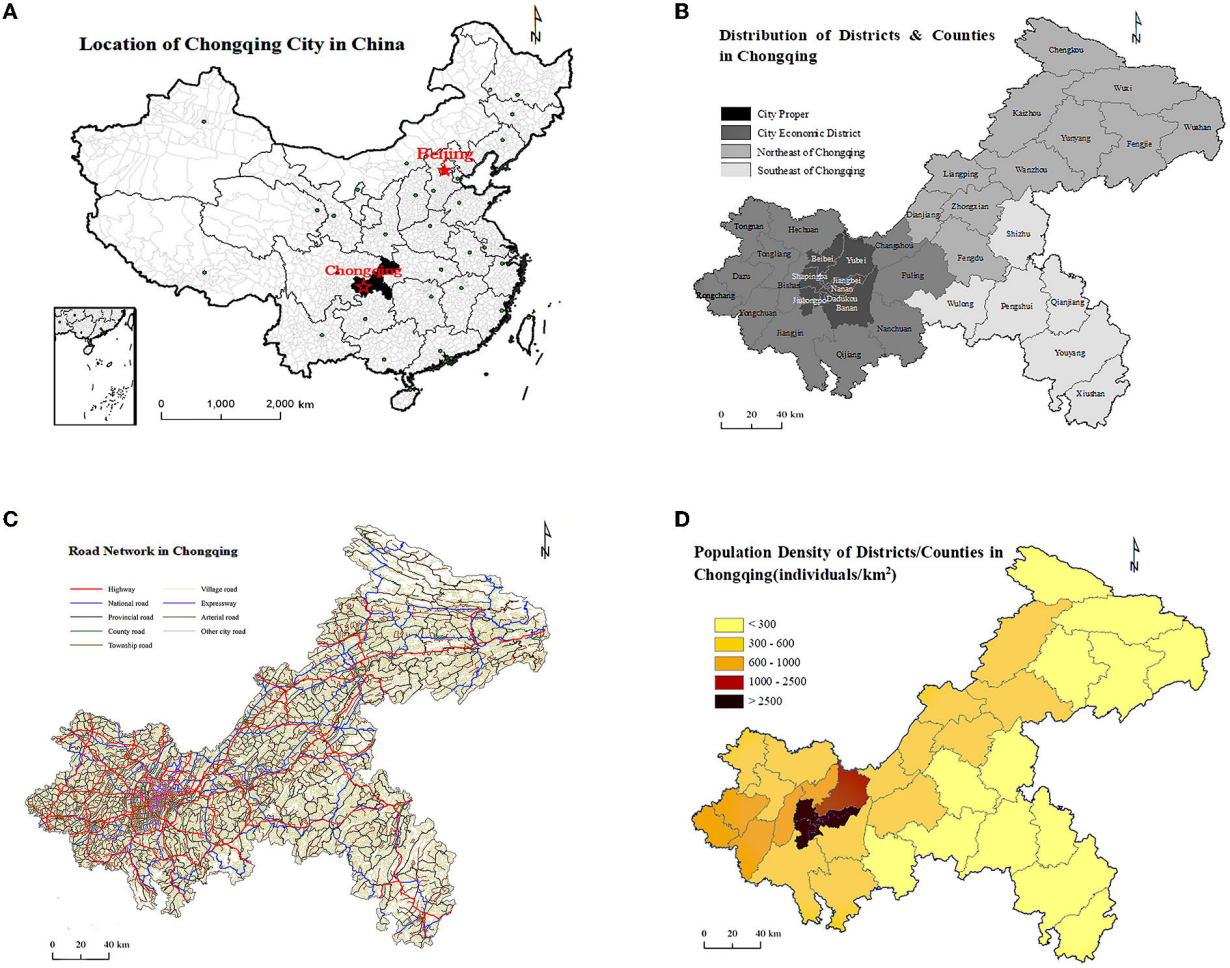
**(A)** Location of Chongqing in China. **(B)** Distribution of districts and countries in four economic zones. **(C)** Road network. **(D)** Population density of Chongqing, 2020.

Chongqing is located in the southwest of China, with a total area of 82,400 square kilometers mainly mountainous. Chongqing has 38 districts/counties and a total population of 31.1 million by the end of 2020 (Figure 1). According to the level of economic development and geographical location, Chongqing is divided into four economic zones. The city proper (including nine districts such as Yuzhong District) and city economic district (including 12 districts such as Jiangjin District) are located in the central and western part of Chongqing, with relatively flat terrain and a more developed economy and a higher urbanization rate. The northeast of Chongqing (NEC) (including 11 districts/counties such as Wushan County) and the southeast of Chongqing (SEC) (including Qianjiang, Wulong, and 4 minority autonomous counties), with predominantly mountainous terrain, have low urbanization rates and predominantly rural populations. The population distribution of Chongqing is shown in Figure 1.

### Study setting

We defined the medical facilities that could provide ambulance services as “EMS stations.” In order to reflect the different types of ambulances provided by EMS stations, we divided EMS stations into those equipped with monitoring ambulances and the others. In addition, this study paid more attention to emergency care needed by patients with more serious conditions, so the care facilities only included secondary and above hospitals [hereinafter referred to as the “definitive care facilities (DCF)”]. Based on the above classification, the first travel time interval (get ambulance service) was divided into 2 types: (i) the nearest EMS station to the settlement; (ii) the nearest EMS station equipped with monitoring ambulances to the settlement. The second travel time interval (get definitive care service) was from the settlement to the nearest DCF. Therefore, there were two scenarios for getting complete EMS: (i) the nearest EMS station to the settlement and then to the DCF (hereinafter referred to as “Primary EMS”); (ii) the nearest EMS station equipped with monitoring ambulances to the settlement and then to the DCF (hereinafter referred to as “Advanced EMS”). In this paper, potential accessibility of EMS was focused on the above 2 travel time intervals, which were usually used to estimate potential EMS accessibility (31, 41, 42), and the above 2 complete EMSs could represent the quality of EMS to some extent. The purpose of this paper was to measure spatial accessibility of EMS by calculating the travel times of two ambulance services and two complete EMSs.

### Data collection

The emergency medical institution data in 2020 were obtained from the Chongqing 120 Command and Dispatch Center, which included each institution information (including name, address, class and ambulances types). We included all medical institutions with EMS capacity in 2020, excluding specialty hospitals and institutions with zero EMS trips in 2020, and finally obtained 908 medical institutions.

We acquired the data of settlements from Chongqing Municipal Health Commission. Administrative villages/communities were selected as the basic research unit, and there were 11,139 villages/communities in Chongqing. Considering the mountainous characteristics of Chongqing, we took the administrative centers of the villages/communities as the population centroids, as opposed to simple geographic centroids.

The road network data and administrative boundary data were provided by Chongqing Geographic Information and Remote Sensing Application Center. According to the Technical Standards for Highway Engineering, and in combination with the road type, traffic conditions, physical conditions and other factors, the traffic speeds of all levels of roads were assigned (Table 1).

**Table 1:** Traffic speeds in Chongqing.

**Road types**	**Levels**	**Speeds (km/h)**
Turnpike	Highway	100
	National highway and Provincial highway	60
	County road	40
	Township road	30
City roads	Expressway	70
	Arterial road	50
	Secondary trunk road	40
	Branch road	30
Village roads	Hard surfaced road	30
	Mechanically cultivated road	20
	Trail	5

The geographic area data and average elevation data of each district/county in Chongqing were obtained from the people's government official website of each district/county, and the data of road density, urbanization rate, GDP per capita, road network density and the number of registered physicians were obtained from the Chongqing Statistical Yearbook 2021.

### Study method

#### Nearest neighbor method

In this study, the nearest neighbor method was employed to calculate spatial accessibility by ArcGIS10.7 software. First, we located the settlements, EMS stations and definitive care facilities on the map. Next, we checked the topological relationship of each road and assigned the traffic speeds according to Table 1, and finally constructed a road network dataset. Based on the road network data, the Network Analysis function of ArcGIS software was used to calculate the shortest travel time (from the EMS station to the settlement and from the settlement to the nearest definitive care facility), and the two travel times were added together to obtain the complete shortest travel time. We finally obtained the shortest travel times to get ambulance services and complete EMS for 11,139 settlements.

Considering that the distribution of travel time tended to be skewed, the median and quartiles were used to reveal the central tendency of its variation. To detail the spatial variation in shortest travel times, we divided the shortest travel times for getting ambulance services into five categories ranging from 0 to 5 min to >60 min. The shortest travel time to get complete EMS was divided into six categories ranging from 0 to 10 min to >120 min, and the percentage of population and community (village) covered by each time interval was presented. For example, the percentage of communities (villages) getting ambulance service with 0–5 min coverage is equal to the number of communities (villages) with the shortest travel time of 0–5 min divided by the total number of communities (villages), while the percentage of population covered is equal to the number of people in communities (villages) with the shortest travel time of 0–5 min divided by the total population. SAS9.4 software was used to conduct a descriptive analysis of EMS institutions and the shortest travel times.

#### Spatial autocorrelation

The median of the shortest travel time of all settlements within each district/county was used to represent the shortest travel time of that district/county, and spatial autocorrelation analysis was performed for the shortest travel time of 38 districts/counties. In this paper, global Moran's I index was used to reveal the overall spatial correlation and agglomeration characteristics (43). The range of Moran's I index is approximately scored from −1 to +1. If the value of I exceeds 0, indicating that the shortest travel time presents spatially positive correlation, the larger the value the more obvious correlation. If the value of I is <0, indicating that the shortest travel time presents spatially negative correlation, the smaller the value the larger the spatial difference. If the value of Moran's I index equals 0, indicating spatially uncorrelated. To further clarify the specific spatial clustering pattern of the shortest travel time, univariate local Moran's I was used to estimate the degree of significance and spatial difference between each study area and its surrounding areas (44). The specific spatial locations where the clustering relationships occurred and the clustering patterns were presented by local indicators of spatial association (LISA), including High-High Cluster, High-Low Outlier, Low-Low Cluster and Low-High Outlier. The spatial autocorrelation was analyzed by GeoDa1.13.

#### Correlation and regression

Socioeconomic, medical resources, road network, geographical and population factors have been shown to influence the travel time of residents to get health care resources (18, 20, 21, 24, 45, 46). EMS, as a public health resource (33, 39), may be similarly influenced. In this study, socioeconomic factors (urbanization rate and GDP per capita), healthcare resources (density of secondary and above EMS hospitals and the number of registered physicians), road network density, geographical factor (average elevation) and population factor (number of settlements) were selected. Pearson correlation was used to analyze the degree of correlation between the above factors and the shortest travel time of districts/counties, where the shortest travel time of each district/county was expressed by the median of the shortest travel time of all settlements within each district/county. Multiple linear stepwise regression analysis was conducted with the shortest travel time of the district/county as the dependent variable and other factors as independent variables. The correlation and regression analysis were conducted by SAS9.4 software.

## Results

### Description analysis of EMS institutions

**Figure 2:**
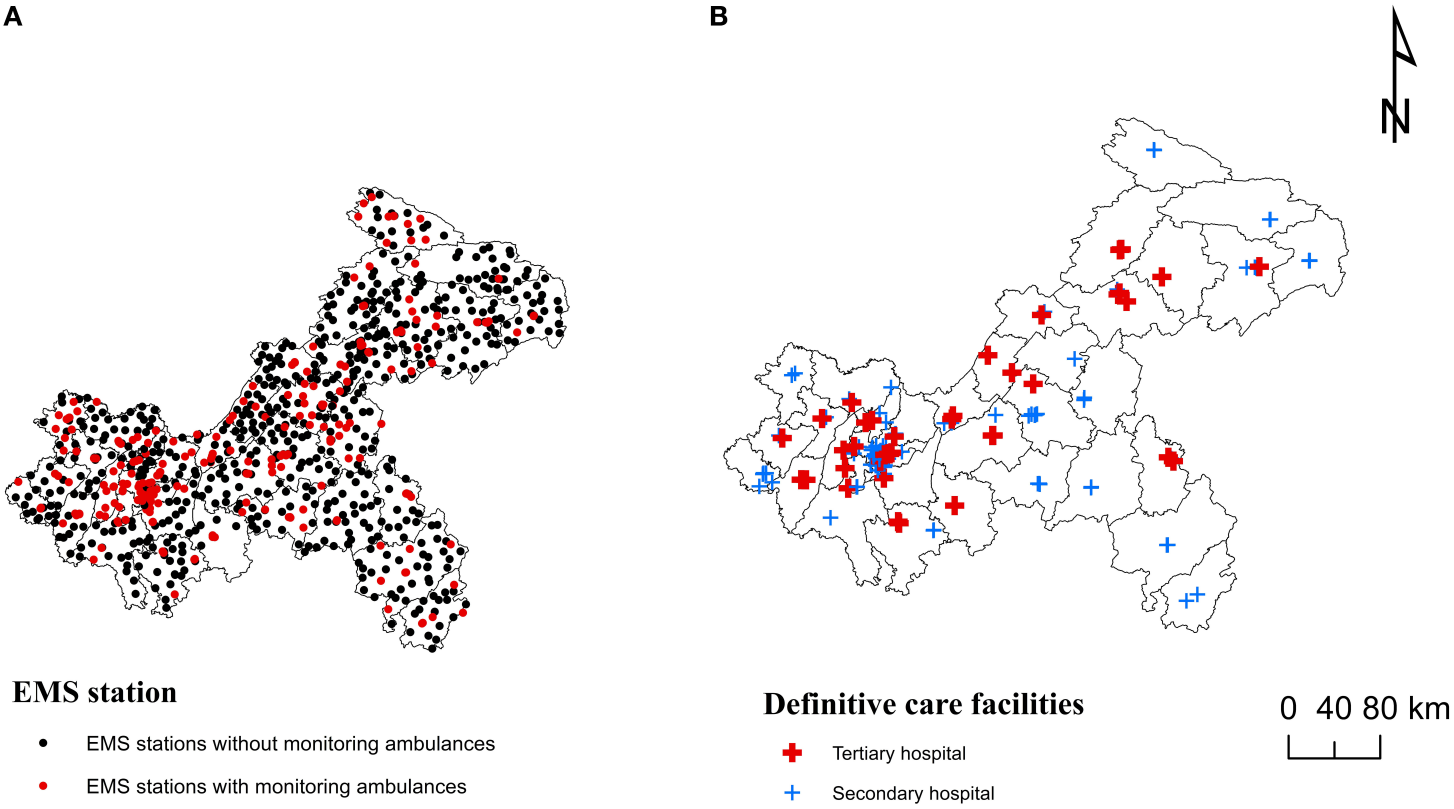
Spatial distribution of emergency medical services (EMS) institutions in Chongqing: **(A)** EMS stations. **(B)** Definitive care facilities.

The 908 EMS institutions were mainly primary health institutions, accounting for 79.5%, and 152 EMS institutions were secondary and above hospitals, accounting for 16.7% (Table 2). The city proper had the highest percentage of secondary and above hospitals, accounting for 35.5%, while there were few in northeast and southeast Chongqing, accounting for 23.0 and 11.2%, respectively. Less than one-third of EMS stations had monitoring ambulances, and the distribution was uneven between four regions. More than half (56.2%) of the EMS stations in CP had monitoring ambulances, while there was only 19.0% in NEC (Table 2). Figure 2 showed the spatial distribution of EMS stations and definitive care facilities.

**Table 2:** Distribution of EMS institutions in Chongqing.

	**EMS institutions** ***n*** **(%)**					**EMS stations with and without monitoring ambulances** ***n*** **(%)**		

**Regions**	**Primary health institutions**	**Grade-one hospitals**	**Secondary hospitals**	**Tertiary hospitals**	**Total**	**With**	**Without**	**Total**
Chongqing	722 (79.5)	34 (3.7)	111 (12.2)	41 (4.5)	908 (100.0)	244 (26.9)	664 (73.1)	908 (100.0)
CP	37 (35.2)	14 (13.3)	39 (37.1)	15 (14.3)	105 (100.0)	59 (56.2)	46 (43.8)	105 (100.0)
CED	188 (77.1)	10 (4.1)	31 (12.7)	15 (6.1)	244 (100.0)	73 (29.9)	171 (70.1)	244 (100.0)
NEC	338 (88.0)	11 (2.9)	26 (6.8)	9 (2.3)	384 (100.0)	73 (19.0)	311 (81.0)	384 (100.0)
SEC	152 (86.9)	6 (3.4)	15 (8.6)	2 (1.1)	175 (100.0)	39 (22.3)	136 (77.7)	175 (100.0)

CP, the city proper; CED, the city economic district; NEC, the northeast of Chongqing; SEC, the southeast of Chongqing; EMS, emergency medical services.

### Spatial accessibility of EMS

#### Spatial accessibility of ambulance services

**Figure 3:**
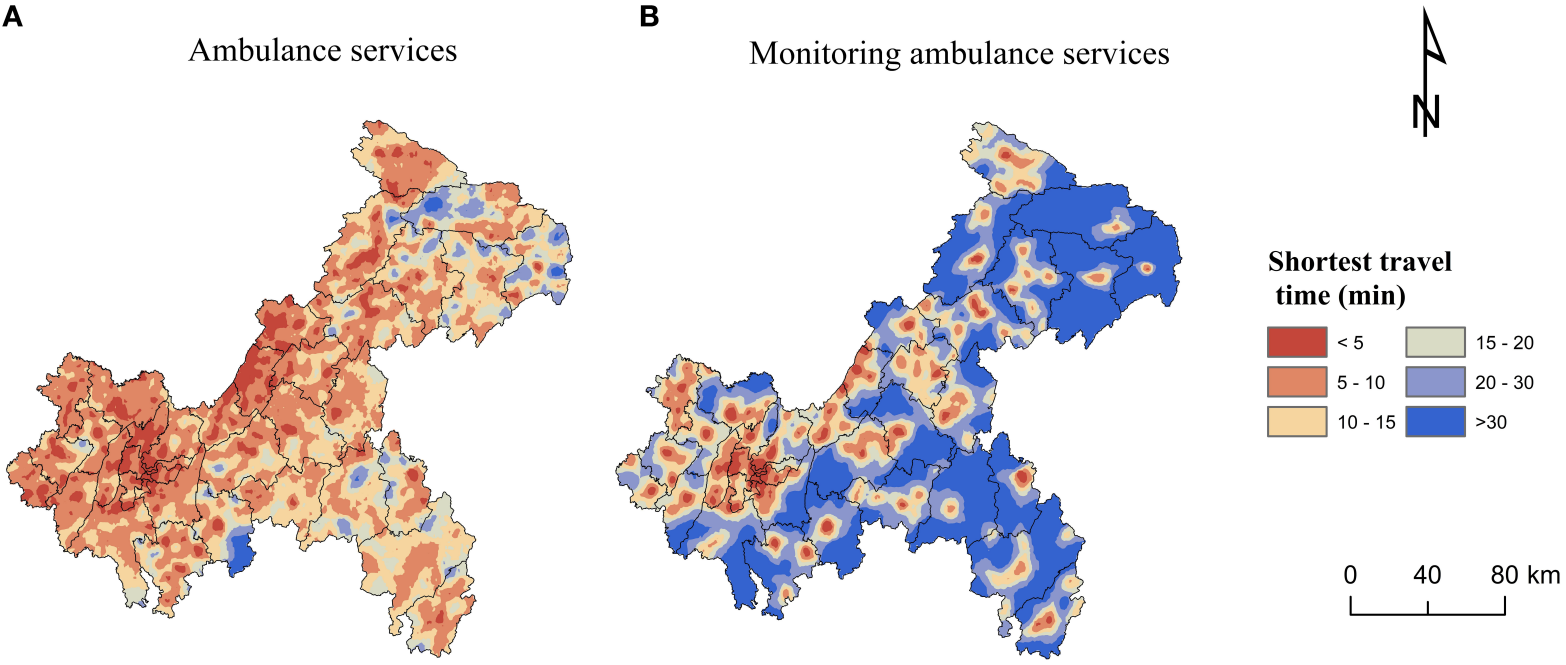
Spatial accessibility of ambulance services: **(A)** Ambulance services. **(B)** Monitoring ambulance services.

The median of shortest travel time to get ambulance services in Chongqing was 7.0 min, of which the travel time in CP was the shortest, with a median of 3.6 min, and that in SEC was the longest, with a median of 9.6 min. 89.5% of the population and 81.5% of the communities/villages in Chongqing were able to get ambulance services within 15 min, and only 79.7% of the population and 70.8% of the communities/villages in SEC were able to get them within 15 min (Table 3). Figure 3A showed a more detailed spatial distribution of the shortest travel times for the settlements to get ambulance services.

**Table 3:** Distribution of the shortest ravel time to get ambulance services.

	**Shortest travel time (min)**	**Percentage of covered population (communities/villages) at different shortest travel times**				
**Regions**	**Median (P_25_-P_75_)**	**[0, 5) min**	**[5, 10) min**	**[10, 15) min**	**[15, 30) min**	**[30, ~) min**
Chongqing	7.0 (2.6–12.9)	56.9 (39.3)	20.8 (24.9)	11.8 (17.3)	9.4 (16.1)	1.1 (2.4)
CP	3.6 (1.7–7.9)	80.4 (61.5)	14.0 (21.4)	4.3 (11.9)	1.3 (5.2)	0.0 (0.0)
CED	7.3 (3.0–12.4)	49.1 (35.5)	25.6 (29.2)	15.6 (19.8)	9.0 (14.0)	0.7 (1.5)
NEC	7.7 (2.8–14.3)	49.5 (36.6)	21.5 (23.8)	12.8 (16.7)	14.1 (19.0)	2.1 (3.9)
SEC	9.6 (3.9–16.5)	44.2 (30.1)	19.9 (21.7)	15.6 (19.0)	17.8 (25.5)	2.5 (3.7)

CP, the city proper; CED, the city economic district; NEC, the northeast of Chongqing; SEC, the southeast of Chongqing.

The median of shortest travel time to get monitoring ambulance services was 18.6 min. The median of shortest travel time in CP was 5.5 min, which was not much different from the time to get ambulance services. The median of shortest travel time in SEC was 26.4 min, which was nearly three times longer than the time to get ambulance services. 82.0% of the population and 71.5% of the communities/villages in Chongqing were able to get monitoring ambulance services within 30 min, while only 66.7% of the population and 57.3% of the communities/villages in SEC were able to get them within 30 min (Table 4). Figure 3B showed a more detailed spatial distribution of the shortest travel times for the settlements to get monitoring ambulance services.

**Table 4:** Distribution of the shortest ravel time to get monitoring ambulance services.

	**Shortest travel time (min)**	**Percentage of covered population (communities/villages) at different shortest travel times**					

**Regions**	**Median (P**_**25**_ **-P**_**75**_ **)**	**[0, 5) min**	**[5, 10) min**	**[10, 15) min**	**[15, 30) min**	**[30, 60) min**	**[60**, ~**) min**
Chongqing	18.6 (8.2–32.4)	38.0 (18.0)	11.8 (11.4)	9.6 (11.7)	22.6 (30.4)	15.5 (23.5)	2.5 (5.0)
CP	5.5 (2.4–14.4)	71.8 (48.0)	12.6 (15.6)	6.0 (12.5)	7.8 (18.2)	1.8 (5.6)	0.0 (0.1)
CED	16.3 (8.5–25.7)	30.0 (15.6)	14.3 (14.0)	14.3 (16.0)	28.6 (36.5)	12.0 (16.3)	0.8 (1.6)
NEC	25.5 (13.3–51.5)	22.9 (10.8)	9.2 (8.6)	7.9 (8.5)	27.1 (30.2)	26.8 (32.3)	6.1 (9.6)
SEC	26.4 (14.2–40.7)	21.9 (9.6)	8.6 (7.7)	8.4 (9.4)	27.8 (30.6)	29.2 (37.0)	4.1 (5.7)

CP, the city proper; CED, the city economic district; NEC, the northeast of Chongqing; SEC, the southeast of Chongqing.

#### Spatial accessibility of complete EMS

**Figure 4:**
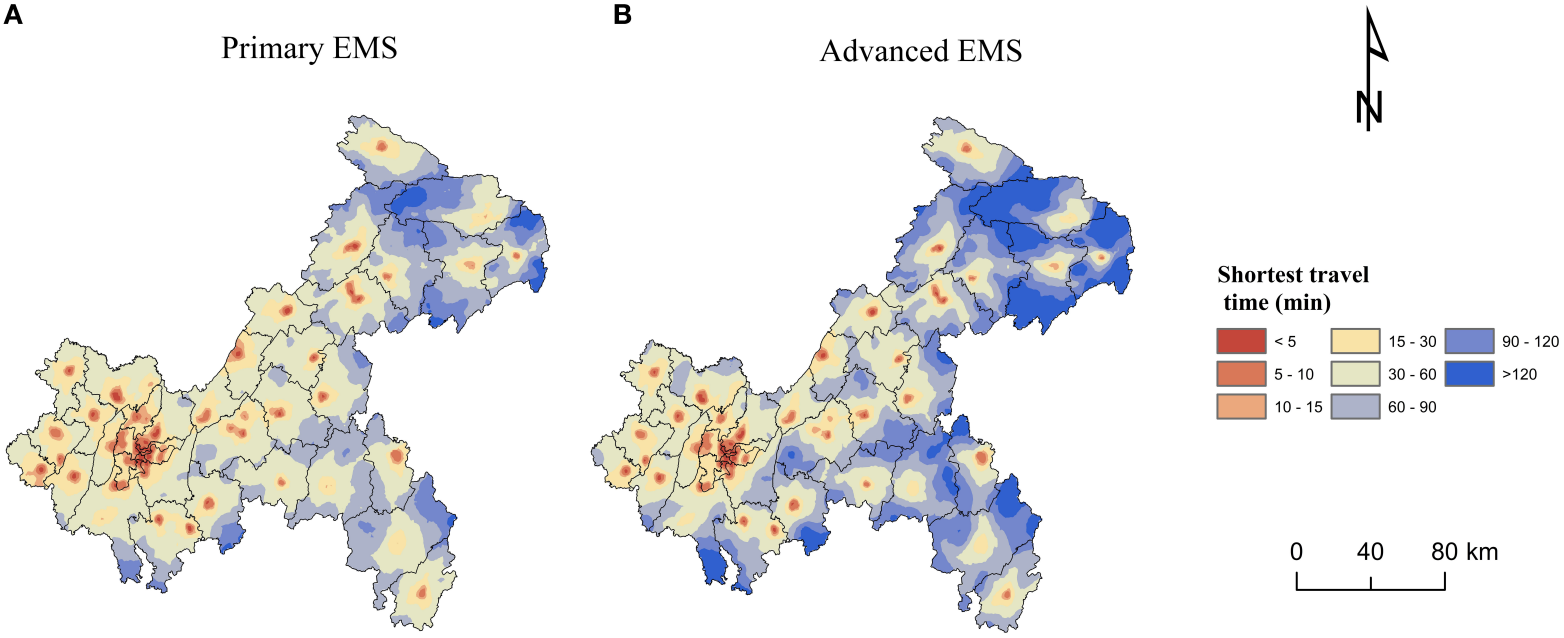
Spatial accessibility of complete emergency medical services (EMS): **(A)** Primary EMS. **(B)** Advanced EMS.

The median of shortest travel time to get primary EMS in Chongqing was 36.2 min. The median of shortest travel time in CP was 11.3 min and in SEC was 51.7 min. 99.2% of the population and 97.0% of communities/villages in CP could get primary EMS within 1 h, while only 70.6% and 60.9% in SEC (Table 5). Figure 4A showed a more detailed spatial distribution of the shortest travel times for the settlements to get primary EMS.

**Table 5:** Distribution of the shortest ravel time to get primary emergency medical services (EMS).

	**Shortest travel time (min)**	**Percentage of covered population (communities/villages) at different shortest travel times**					

**Regions**	**Median (P**_**25**_ **-P**_**75**_ **)**	**[0, 10) min**	**[10, 30) min**	**[30, 60) min**	**[60, 90) min**	**[90, 120) min**	**[120**, ~**) min**
Chongqing	36.2 (19.1–56.0)	35.4 (14.8)	25.1 (25.7)	27.3 (38.2)	8.8 (14.7)	2.7 (4.9)	0.7 (1.62)
CP	11.3 (4.8–26.3)	71.1 (46.1)	22.5 (32.5)	5.6 (18.4)	0.8 (3.0)	0.0 (0.0)	0.0 (0.0)
CED	30.9 (19.4–43.4)	26.4 (11.5)	35.2 (36.1)	33.0 (43.8)	4.5 (6.8)	0.7 (1.3)	0.2 (0.5)
NEC	47.8 (29.7–69.0)	20.6 (8.1)	18.5 (17.5)	36.8 (41.0)	16.4 (21.4)	6.0 (8.8)	1.7 (3.2)
SEC	51.7 (33.8–72.6)	17.4 (5.5)	17.4 (15.8)	35.8 (39.6)	22.1 (28.8)	6.2 (8.6)	1.1 (1.7)

CP, the city proper; CED, the city economic district; NEC, the northeast of Chongqing; SEC, the southeast of Chongqing.

The median of shortest travel time to get advanced EMS in Chongqing was 47.8 min (Table 6), which was longer than getting primary EMS (Table 5). The median of shortest travel time in CP was 13.8 min and in SEC was 68.5 min. 97.5% of the population and 91.2% of the communities/villages in CP were able to get advanced EMS within 1 h, while <50% of the population and communities/villages were able to get advanced EMS within 1 h in SEC and NEC, where nearly 10% of the population and more than 10% of the communities/villages were unable to get advanced EMS within 2 h (Table 6). Figure 4B showed a more detailed spatial distribution of the shortest travel times for the settlements to get advanced EMS.

**Table 6:** Distribution of the shortest ravel time to get advanced emergency medical services (EMS).

	**Shortest travel time (min)**	**Percentage of covered population (communities/villages) at different shortest travel times**					

**Regions**	**Median (P**_**25**_ **-P**_**75**_ **)**	**[0, 10) min**	**[10, 30) min**	**[30, 60) min**	**[60, 90) min**	**[90, 120) min**	**[120**, ~**) min**
Chongqing	47.8 (23.2–76.6)	33.8 (13.6)	18.5 (17.4)	23.8 (31.4)	13.6 (20.0)	6.6 (10.6)	3.7 (7.0)
CP	13.8 (5.1–33.7)	68.8 (43.5)	21.2 (28.5)	7.5 (19.2)	2.2 (7.7)	0.3 (1.0)	0.0 (0.1)
CED	39.1 (23.4–58.1)	25.1 (10.5)	24.9 (24.0)	33.8 (42.6)	11.6 (16.1)	3.7 (5.0)	0.9 (1.8)
NEC	64.9 (40.2–96.0)	18.6 (6.8)	11.3 (9.8)	26.8 (28.7)	22.2 (25.4)	12.5 (16.2)	8.6 (13.1)
SEC	68.5 (43.6–97.5)	17.0 (5.3)	11.6 (10.0)	24.2 (25.9)	24.6 (28.3)	14.8 (19.8)	7.8 (10.7)

CP, the city proper; CED, the city economic district; NEC, the northeast of Chongqing; SEC, the southeast of Chongqing.

**Figure 5:**
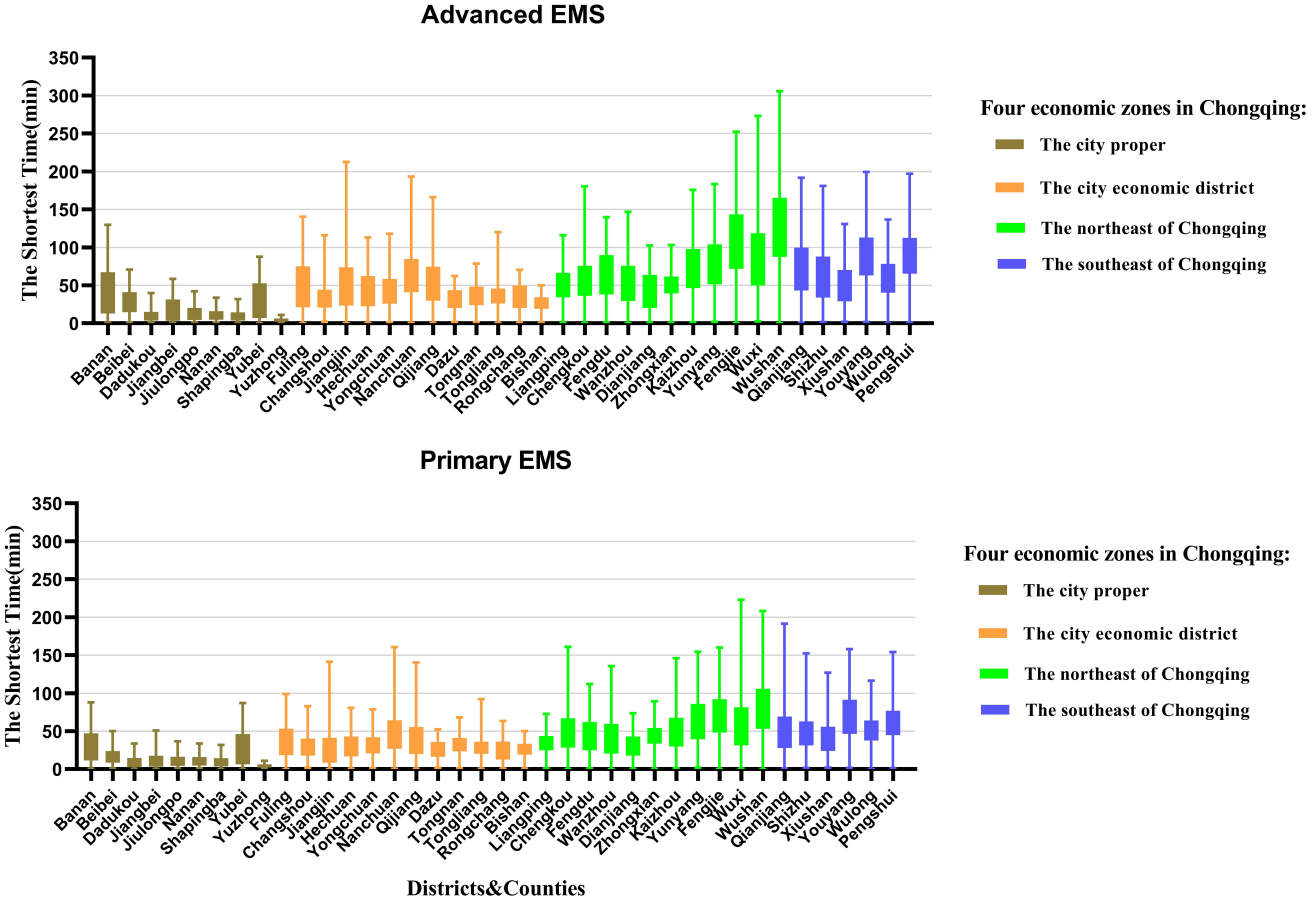
Distribution of the shortest travel time to get primary and advanced emergency medical services (EMS) for each settlement in 38 districts/counties in Chongqing.

Figure 5 presented the distribution of the shortest travel time to get primary and advanced EMS for each settlement in 38 districts/counties, with large differences in the time distribution among the districts/counties. Yuzhong District, located in CP, had the shortest medians of time to get primary and advanced EMS, both of which were 4.0 min with no difference between them. While Wushan County in NEC had the longest time, the medians of time to get primary and advanced EMS were 74.3 and 120.8 min, respectively, with a large difference. Within each district/county, there were also differences in EMS travel times. There was little difference within each district in CP. For example, the interquartile ranges of primary and advanced EMS time in Yuzhong District were both 2.8 min. However, there was a large variation within districts/counties in SEC and NEC, especially the time for getting advanced EMS. For example, the interquartile range of time to get primary EMS in Wushan County was 52.5 min, while the time to get advanced EMS was 77.7 min.

### Spatial autocorrelation analysis

**Figure 6:**
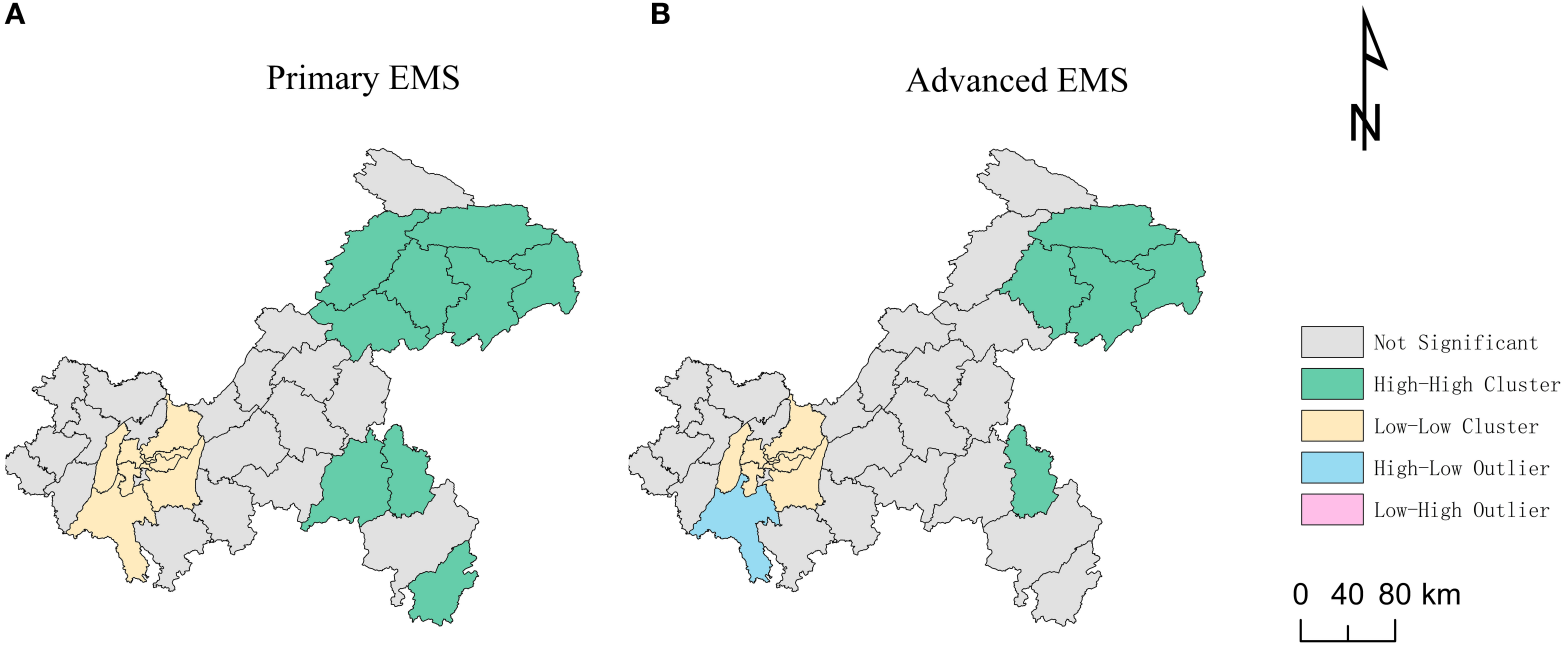
Local indicators of spatial association (LISA) of the shortest travel times: **(A)** Primary emergency medical services (EMS). **(B)** Advanced EMS.

The global autocorrelation analysis of the shortest travel times for primary and advanced EMS indicated that the Moran's I indexes were 0.743 and 0.770, respectively, with *P*-values of 0.0010 which passed the significance test. The results indicated an agglomeration characteristic, and there was a positive spatial correlation in the travel time of primary and advanced EMS in Chongqing. As shown in Figure 6, the EMS travel time of the study area showed obvious agglomeration of high-value regions and low-value regions. The results of local indicators of spatial association (LISA) were similar. The Low-Low types that referred to cluster of short EMS travel time mainly distributed in city proper. The High-High types that referred to cluster of long EMS travel time mainly distributed in northeast and southeast of Chongqing.

### Correlation and regression analysis

#### Pearson correlation analysis

The shortest travel times for primary and advanced EMS had a negative correlation with urbanization rate, GDP per capita, the density of secondary and above EMS hospitals, the number of registered physicians and road network density; while them had a positive correlation with average elevation and the number of settlements. The shortest travel times for primary and advanced EMS had the highest correlation coefficient with urbanization rate, followed by GDP per capita and average elevation (Table 7).

**Table 7:** Pearson's correlation results of shortest travel times for primary and advanced emergency medical services (EMS).

**The shortest travel times**	**Urbanization rate**	**Log of GDP**	**Density of secondary and above EMS hospitals**	**Number of registered physicians**	**Road network density**	**Average elevation**	**Number of settlements**
Primary EMS	−0.850^**^	−0.746^**^	−0.435^**^	−0.609^**^	−0.442^**^	0.732^**^	0.517^**^
Advanced EMS	−0.814^**^	−0.728^**^	−0.397^*^	−0.572^**^	−0.408^*^	0.718^**^	0.507^**^

** 1% significance levels;

* 5% significance levels.

EMS, emergency medical services.

#### Stepwise regression analysis

The above factors for correlation analysis were included in the stepwise regression analysis. The VIF values of all variables were below 3, indicating that there was no serious covariance in the regression model. The number of settlements and average elevation were positive influencing factors on the shortest travel time for primary EMS, while the urbanization rate was a negative influencing factor. The number of settlements was a positive influencing factor on the shortest travel time for advanced EMS, and GDP per capita and urbanization rate were negative influencing factors (Table 8).

**Table 8:** Stepwise regression results of shortest travel times for primary and advanced emergency medical services (EMS).

**The shortest times**	**Variables**	**Coefficient**	***P***	**VIF**	**Adjusted *R*^2^**
Primary EMS	Urbanization rate	−0.519	0.000	2.135	0.804
	Average elevation	0.020	0.001	1.825	
	Number of settlements	0.037	0.005	1.233	
Advanced EMS	Urbanization rate	−0.783	0.000	2.030	0.732
	Log of GDP	−21.270	0.009	1.844	
	Number of settlements	0.047	0.040	1.207	

EMS, emergency medical services.

## Discussion

Understanding the time impedance of residents to get EMS was conducive to assessing the spatial accessibility of EMS in a wider range of areas and comparing their geospatial differences. This was conducive to better allocation of EMS resources and planning of health care in the future.

Our findings indicated that the median of shortest travel time to get ambulance service in Chongqing was 7.0 min, with about 90% of the population able to get ambulance services within 15 min and about 99% of the population able to get ambulance services within 30 min, which was better than Ghana (26) and southern Sweden (47), showing a relatively high accessibility. This was related to the vigorous construction of EMS systems in recent years. In 2019, Chongqing Municipality issued Chongqing pre-hospital emergency network medical institution management measures (48), relying on all levels of hospitals and primary medical institutions to build a pre-hospital emergency network system covering urban and rural areas, which greatly improved the EMS accessibility for urban and rural residents. In particular, the “dependent” mode was adopted to incorporate rural township health centers into EMS system, establish EMS stations, allocate ambulances, and train health personnel in emergency medical rescue, which not only solved the problem of setting up EMS stations in the vast rural areas with relatively low population density, but also effectively solved the problem of insufficient human resources, greatly shortening the time for rural residents to obtain EMS.

However, the study also showed that accessibility of higher quality ambulance services was relatively poor. The median of the shortest travel time for monitoring ambulance services was 18.6 min, much longer than the shortest travel time for getting ambulance service. This was related to inadequate configuration and uneven distribution of monitoring ambulances. Due to economic constraints, the primary medical institutions in rural areas were usually equipped with transit-type ambulances (49). The results of our study showed that more than 70% of EMS stations in Chongqing were not equipped with monitoring ambulances. It was difficult to provide the necessary prehospital emergency care for emergency and critical patients with simple and crude equipment of transit-type ambulances (36), such as failure to provide essential defibrillators and ventilators for patients in cardiac arrest. Therefore, increasing the allocation of monitoring ambulances in primary care facilities could improve rural residents to get advanced EMS, which could greatly contribute to reducing the mortality rate of critically injured patients.

Our results also emphasized the integration of two relevant trips to measure accessibility of EMS. The “dependent” mode was adopted to establish EMS stations by relying on widely distributed primary care facilities, so that ambulances could reached residents in a short period of time and the accessibilities of ambulance service and complete EMS which integrated two related trips were increased. The results of our study showed that more than 80% of population could get complete primary EMS within “golden hour,” which also met the WHO initiative that more than 80% of population should get emergency medical services within 2 h by 2030 (6). However, the accessibility of complete EMS was unevenly distributed due to the limited number and uneven distribution of secondary and higher medical facilities. The univariate spatial autocorrelation results showed that the Low-Low types that referred to cluster of short EMS travel time mainly distributed in city proper. The accessibility of complete EMS in the city proper had obvious advantages, with the median of the shortest travel time of only 11.3 min, which was better than that in the central city of Wuhan (32). The city proper was developed area in Chongqing with dense population distribution, well-developed road networks, and high investment in healthcare resources. Our survey results showed that more than 35% of secondary and above EMS hospitals were concentrated in city proper. High-High types that referred to cluster of long EMS travel time mainly distributed in southeast and northeast of Chongqing. Most of these areas were rural areas with relatively low population density, and the number of secondary and above hospitals was low such as only 11.2% in southeast of Chongqing. The time to get complete EMS in southeast and northeast of Chongqing was about five times longer than that of the city proper. The EMS accessibility in remote communities/villages far from hospitals was even worse. The results of the study showed that nearly 10% of the population and more than 10% of the communities/villages in southeast and northeast of Chongqing could not get advanced EMS within 2 h.

This study explored the relationship between the shortest travel time of complete EMS and socio-economic, healthcare resource, geographical, and population factors. The results showed that urbanization rate, GDP per capita, the density of secondary and above EMS hospitals, the number of registered physicians, average elevation, road network density and the number of settlements were correlated with spatial accessibility of EMS. The stepwise regression results indicated that settlements in the regions with high urbanization rates were more likely to get complete EMS, while settlements in the regions with high GDP per capita were more likely to get advanced EMS. This was because areas with high urbanization and economic development invested more in healthcare resources and had adequate allocations of high-quality healthcare resources (18, 49). The survey results showed that more than half of EMS stations in city proper had monitoring ambulances, while only 19.0% in the northeast of Chongqing; More than 35% of secondary and above hospitals were located in city proper, while only 23.0% and 11.2% were in northeast and southeast of Chongqing, respectively. In order to narrow the gap between urban and rural areas, and improve the accessibility of EMS for all the people, government departments should strengthen financial investment in the case of EMS in economic backward areas and consider optimizing the number of advanced EMS facilities based on emergency response time and actual population coverage rate (50), such as gradually deploying high-grade EMS equipment in central township health centers in rural areas where conditions permit. In addition, the government should improve the level of emergency care in economic backward areas. For example, it can plan and upgrade the emergency treatment capacity of a minority of central township health centers that are usually located in townships with relatively large populations and have stronger medical capabilities than general township health centers, so that they can gradually reach or approach the level of secondary hospitals, so as to solve the problem of insufficient and uneven distribution of secondary and higher hospitals in rural areas.

In addition, settlements in the areas with high average elevation were more difficult to get primary EMS, which was consistent with the results of previous studies. The poorly developed road network and rugged roads in high-altitude mountainous terrain could lead to longer travel times (20, 21). Therefore, government should plan road construction, increase the density of roads in remote mountainous areas, and upgrade the road grades. The higher number of settlements, the longer EMS travel time. This was because the eastern part of Chongqing was mostly rural area with scattered villages, and the distance between scattered settlements and EMS stations was relatively long (51). Therefore, the management should reasonably plan rural settlements and promote moderate concentration of rural population, which will increase the utilization of healthcare resources.

In recent years, Chongqing has begun to explore the establishment of an air emergency medical rescue system and a water emergency medical rescue system for the Three Gorges Reservoir area such as Wushan County (52). It is believed that a comprehensive EMS system integrating water, land and air can greatly improve the accessibility of EMS in Chongqing. In addition, measures such as strengthening health education, publicizing and popularizing first aid knowledge, developing and training the public's first aid skills, and developing a visual EMS system can greatly compensate for the poor spatial accessibility of EMS due to limited conditions.

### Limitations

There were several limitations in our study. First, the nearest neighbor method was a clear and easily explained concept, with shorter travel times indicating better accessibility, which was easily communicated to policymakers (14, 20). However, it lacked in considering actual health care utilization patterns, such as the service capacity of medical facilities and patient choice. Second, we used the network analysis function of ArcGIS to calculate the shortest travel time without considering the actual road conditions such as traffic congestion and traffic light waiting time, which might deviate from the real travel time. Therefore, in future research, we will consider more actual road conditions to more accurately evaluate the spatial accessibility of EMS.

## Conclusion

This paper analyzed the accessibility of EMS in Chongqing, China, from the perspective of potential spatial accessibility. This paper also explored its influencing factors and regional differences. By adopting the “dependent” mode, the ambulance accessibility was relatively high, but the accessibility of monitoring ambulance was relatively low. Urban-rural differences in accessibility of complete EMS which integrated two related trips were obvious. The accessibilities of primary and advanced EMS in the city proper and the city economic district were much higher than that in southeast and northeast of Chongqing. This difference was mainly attributed to socioeconomic, healthcare resources and geographical factors. Therefore, relevant policy makers should pay special attention to areas with poor accessibility of EMS. It is recommended to increase financial investment in EMS for these areas, increase high-quality EMS resources, upgrading EMS capacity of central township health centers, strengthen road construction in mountainous areas, and provide reasonably plan rural settlements for reducing disparity between regions and improving the equity in getting EMS for all the people. Our findings will help policy makers improve spatial accessibility of EMS in Chongqing and provide a basis for evaluating of spatial accessibility of EMS and optimizing allocation of EMS resources in economic backward countries and regions. Although this study was conducted in Chongqing, China, the approach of integrating two travel trips involved in EMS, considering ambulance types, and stratifying analysis by EMS resource levels was also applicable to other similar studies.

## Data availability statement

The data analyzed in this study is subject to the following licenses/restrictions: The data analyzed in this study were obtained from the Chongqing 120 Command and Dispatch Center, Chongqing Municipal Health Commission and Chongqing Geographic Information and Remote Sensing Application Center, but restrictions apply to the availability of these data, which were used under license for the current study, and so are not publicly available. Data are however available from the authors upon reasonable request and with permission of the above institutions. Requests to access these datasets should be directed to DX, xdgtxm@163.com.

## Author contributions

YZh, DX, YZo, and LJ designed the study. DX, LJ, YZo, XD, ZC, YH, SC, and QW collected the data. YZo, LJ, and SC performed the statistical analysis. YZo, YZh, and LJ wrote the manuscript. XD, ZC, YH, SC, QW, DX, and YZh helped to draft the manuscript. All authors have read and approved the final manuscript.
